# Supervised progressive cross-continuum strength training compared with usual care in older medical patients: study protocol for a randomized controlled trial (the STAND-Cph trial)

**DOI:** 10.1186/s13063-016-1309-1

**Published:** 2016-04-01

**Authors:** Mette Merete Pedersen, Janne Petersen, Nina Beyer, Lars Damkjær, Thomas Bandholm

**Affiliations:** Optimed, Clinical Research Centre and Physical Medicine Research-Copenhagen (PMR-C), Department of Physiotherapy, Copenhagen University Hospital, Hvidovre, Denmark; Optimed, Clinical Research Centre, Copenhagen University Hospital, Hvidovre, Denmark; Section of Biostatistics, Department of Public Health, University of Copenhagen, Copenhagen, Denmark; Musculoskeletal Rehabilitation Research Unit, Bispebjerg and Frederiksberg Hospitals, University of Copenhagen, Copenhagen, Denmark; Department of Clinical Medicine, Faculty of Health and Medical Sciences, University of Copenhagen, Copenhagen, Denmark; Department of Rehabilitation, Copenhagen Municipality Health Administration, Copenhagen, Denmark; Optimed, Clinical Research Centre and Physical Medicine Research-Copenhagen (PMR-C), Clinical Research Centre, Copenhagen University Hospital, Hvidovre, Denmark; Department of Orthopaedic Surgery, Copenhagen University Hospital, Hvidovre, Denmark

**Keywords:** Older medical patients, Hospitalization, Progressive strength training, Supervision, Mobility, Cross-continuum

## Abstract

**Background:**

Hospitalization in older adults is characterized by physical inactivity and a risk of losing function and independence. Systematic strength training can improve muscle strength and functional performance in older adults. Few studies have examined the effect of a program initiated during hospitalization and continued after discharge. We conducted a feasibility study prior to this trial and found a progression model for loaded sit-to-stands feasible in older medical patients. This study aims to determine whether a simple supervised strength training program for the lower extremities (based on the model), combined with post-training protein supplementation initiated during hospitalization and continued at home for 4 weeks, is superior to usual care on change in mobility 4 weeks after discharge in older medical patients.

**Methods:**

Eighty older medical patients (65 years or older) acutely admitted from their own homes will be included in this randomized, controlled, parallel-group, investigator-blinded, superiority trial. After baseline assessments patients will be randomized to (1) intervention: progressive strength training during hospitalization and after discharge (home-based), or (2) control: usual care. Shortly after discharge, 4 weeks after discharge (primary end point) and 6 months after discharge patients will be assessed in their own homes. The intervention encompasses strength training consisting of two lower extremity exercises (sit-to-stand and heel raise) daily during hospitalization and three times per week for 4 weeks after discharge. Both exercises follow pre-defined models for progression and will be performed for three sets of 8–12 repetitions maximum in each training session. Thereafter, the patient will be asked to consume a protein supplement given orally containing 18 g milk-based protein. The primary outcome will be change in the de Morton Mobility Index score from baseline to 4 weeks after discharge. Secondary outcomes will be 24-h mobility level, isometric knee extension strength, the 30-sec sit-to-stand test, habitual gait speed, hand-grip strength, and Activities of Daily Living.

**Discussion:**

We chose to investigate the effect of a minimal time-consuming treatment approach, i.e. two well-performed strength training exercises combined with protein supplementation, to facilitate implementation in a busy clinical care setting, given a positive trial outcome.

**Trial registration:**

ClinicalTrials.gov: NCT01964482.

## Background

### Background and rationale

Ageing is associated with a decline in muscle strength and functional performance, which is why older adults (aged 65 years or older) do not possess the same reserve capacity as younger adults [1–5]. In general, older hospitalized adults display poor muscle strength and functional performance indicative of poor mobility [6, 7] and are at risk of becoming dependent after acute illness and hospitalization [8–10]. Moreover, hospitalization is associated with a subsequent loss of muscle strength [11], putting hospitalized older adults at a higher risk of losing independence as a consequence of their hospitalization.

During hospitalization, older adults spend most of their time being physically inactive and lying in bed [12–16]. This can lead to a decline in observed and self-reported ability to perform Activities of Daily Living (ADL) at discharge and at 1 month follow-up [14, 17], inducing a risk of dependency [18], and increasing the risk of institutionalization and of death [14]. Older adults are more sensitive to bed rest inactivity compared to younger adults [19–22], and have an impaired ability to fully recover [20, 21]. In healthy older adults, restricted activity and bed rest are associated with reduced protein synthesis and reduced muscle mass and strength [21, 23, 24], and new disabilities in ADL [25, 26]. Similarly, a study by Boyd et al. [9] has shown that new disabilities in ADL are experienced by one third of older medical patients from hospital admission to discharge, and only 30 % of these return to their preadmission level within the first year after discharge [9]. Self-reported decline is seen even after short hospital stays [27]. Thus, reducing physical inactivity during hospitalization and maintaining independency, is considered the most important health outcome by many older adults [28]. Regaining function within the first month after discharge seems especially important as 1-month status is indicative of functional status 1 year after discharge [9].

Systematic strength training can improve muscle strength and functional performance in healthy older adults [29–32], and this has also been reported in patients with chronic diseases [33]. Both strength training initiated during hospitalization in geriatric patients [34], as well as post-discharge training [35, 36] and training of functionally impaired community-dwelling older adults [37], have shown positive effects on strength and functional performance. Most exercise programs for older hospitalized [36, 38–40] or community-dwelling [36, 37, 41–43] adults cover a range of exercises, including upper body and lower body strength training, balance exercises, walking exercises and stretching exercises, but few have examined the effect of a program initiated during hospitalization and continued after discharge [36, 40, 44]. These studies, however, have experienced problems with compliance [36, 40, 44]. A recent systematic review suggests that “the recovery of patients could further benefit from a community-based or an in-home intervention program which build on in-hospital programs” [45]. In addition, acutely hospitalized older adults express the opinion that initiating exercise in the hospital or shortly after discharge is a good idea [44, 46]. Further, supervision can benefit adherence to training [45, 47], and participation is more likely if recommended by a physiotherapist [48]. This emphasizes the importance of supervision by trained staff both in the hospital and in the home setting [40, 44, 49].

Regarding the content of an exercise program, recent reviews suggest that information is lacking about the appropriate dose of strength training in different settings for older adults as well as detailed descriptions of exercises and dosage [29, 50, 51], although it seems that higher intensities are superior to lower intensities [50, 52].

As the lower extremities are especially sensitive to bed rest [23, 53] and lower extremity strength is associated with functional performance (e.g. mobility and the ability to perform ADL) [54–57], it seems reasonable to focus on counteracting loss of strength and functional performance in the lower extremities. Moreover, combining strength training with protein supplementation may be even more beneficial as it may stimulate muscle protein synthesis and thus increase the exercise response on muscle mass and strength as seen in healthy older adults [58–60].

Therefore, the aim of this study is to determine in a randomized, investigator-blinded controlled trial whether a simple, low-technology, supervised strength training program for the lower extremities, combined with post-training protein supplementation initiated during hospitalization and continued at home for 4 weeks after discharge, is superior to usual care on change in mobility 4 weeks after discharge in older medical patients.

## Methods

### Study design

The study, which is called the Cross-Continuum Progressive Strength Training in Older Medical Patients – Copenhagen (STAND-Cph) trial, is a randomized, controlled, parallel-group (two groups), investigator-blinded, superiority trial being conducted in the Copenhagen area, Denmark. The trial investigates the effect of a simple, low-technology, supervised strength training program commenced during hospitalization and continued for 4 weeks after discharge (ClinicalTrials.gov-identifier: NCT01964482). The study is conducted as a full-scale trial following a feasibility study in which we found a progression model for loaded sit-to-stands feasible when used as a simple strength training exercise in older medical patients [61]. Participants will be randomized to either progressive strength training or usual care, and the primary end point will be 4 weeks after discharge (end of exercise period). In addition, the participants will be followed up after 6 months. Table 1 provides an overview of the trial characteristics.

**Table 1:** Trial registration data

Data category	Information
Primary registry and trial identification number	ClinicalTrials.gov: NCT01964482
Data of registration in primary registry	14 October 2013
Secondary identifying numbers	The Ethics Committee of the Capital Region of Denmark: H-2-2012-115
	The Danish Data Protection Agency: 2007-58-0015
Source(s) of monetary or material support	Danish Regions/The Danish Health Confederation, The Lundbeck Foundation (UCSF) (grant numbers FP 07/2012, FP 48/2012 and FP 61/2013), the Research Foundation of Hvidovre Hospital, the Capital Region of Copenhagen, and The Danish Foundation for Research in Physiotherapy
Primary sponsor	Danish Regions/The Danish Health Confederation
Secondary sponsor(s)	The Lundbeck Foundation (UCSF), the Research Foundation of Hvidovre Hospital, the Capital Region of Copenhagen, and The Danish Foundation for Research in Physiotherapy
Contact for public queries	MMP, TB (mette.merete.pedersen@regionh.dk)
Contact for scientific queries	MMP, TB. Clinical Research Centre, Hvidovre Hospital, University of Copenhagen, Denmark
Public title	In-hospital and post-discharge training of older medical patients
Scientific title	Supervised progressive cross-continuum strength training compared with usual care in older medical patients: study protocol for a randomized controlled trial (the STAND-Cph trial)
Country of recruitment	Denmark
Health condition(s) or problem(s) studied	Progressive strength training in older medical patients
Intervention(s)	Intervention: strength training daily during hospitalization and 3 times per week for 4 weeks after discharge
	Control: usual care
Key inclusion and exclusion criteria	Inclusion criteria: age ≥65 years; acutely admitted from own home to the Emergency Department at Hvidovre Hospital, Denmark
	Exclusion criteria: terminal illness; in treatment for a diagnosed cancer; diagnosis of chronic obstructive pulmonary disease (COPD) and participation in a COPD rehabilitation program; living outside the municipalities of Copenhagen and Broendby; inability to speak or understand Danish; inability to cooperate in tests/exercises; an expected hospitalization >24 h; assigned to physical rehabilitation in the community; a Cumulated Ambulation Score (CAS) of 0 in the sit-to-stand item
Study type	Interventional
	Allocation: randomized
	Blinding: investigator blind
Date of first enrollment	September 2013
Target sample size	80
Recruitment status	Recruiting
Primary outcome(s)	The de Morton Mobility Index
	Timeframe: change from baseline to 4 weeks after discharge (end of intervention)
Key secondary outcomes	24-h mobility measured by *activ*PAL3^TM^; isometric knee extension strength in the dominant leg; the 30-sec sit-to-stand test; habitual gait speed; hand-grip strength in dominant hand; the Barthel Index 20

### Study setting

The study will be conducted at Copenhagen University Hospital, Hvidovre, Denmark and in the participants’ own homes in the municipalities of Copenhagen, and Broendby. Hvidovre Hospital has a 552-bed capacity. Recruitment will take place in the 20-bed Emergency Department (ED) through which the majority of older medical patients (65 years or older) are admitted. There are approximately 4000 admissions of older medical patients to the ED every year, and around 50 % are discharged within the first 24 h. In Denmark, the healthcare system is public and provides feeless, tax-paid primary medical care, hospital treatment, and homecare services uniformly for all citizens.

### Study sample and recruitment procedure

Older medical patients (65 years or older) acutely admitted from their own homes to the medical services of the hospital will be included by random sampling within 24 h of admission. Each day (Monday to Friday) the primary investigator or one of three assistant investigators will receive a computer-generated list of all newly admitted older medical patients (65 years or older). The investigator will check the medical records of all the listed patients to determine their eligibility according to the inclusion and exclusion criteria as listed below:

**Inclusion criteria**

- Age 65 years or older
- Admitted from own home to medical services of the hospital

**Exclusion criteria**

- Terminal illness
- In treatment for diagnosed cancer
- Diagnosis of chronic obstructive pulmonary disease (COPD) and participation in a COPD rehabilitation program
- Living outside the municipalities of Copenhagen and Broendby
- Inability to speak or understand Danish
- Inability to cooperate in tests/exercises
- Transferred to the intensive care unit or isolation-room stay
- An expected hospitalization of at least 24 h
- Assigned to physical rehabilitation in the municipality
- A Cumulated Ambulation Score (CAS) of 0 in the sit-to-stand item

Eligible patients will be visited on the ward by one of four investigators where they will be given a written description of the study to read and will be informed about the study verbally. The investigators will ensure that all questions are answered before the patient is asked to participate in the study. The Ethics Committee of the Capital Region has granted an exemption for the 24-h consent time, which is normal practice when including patients for medical research in Denmark. The exemption was granted to be able to follow the patients through their entire hospitalization and to assess their functional level before an effect of medical treatment is seen. Patients who agree to participate will be asked to sign an informed consent form to be included in the study. The patient will keep the original document and two copies will be archived.

**Fig. 1:**
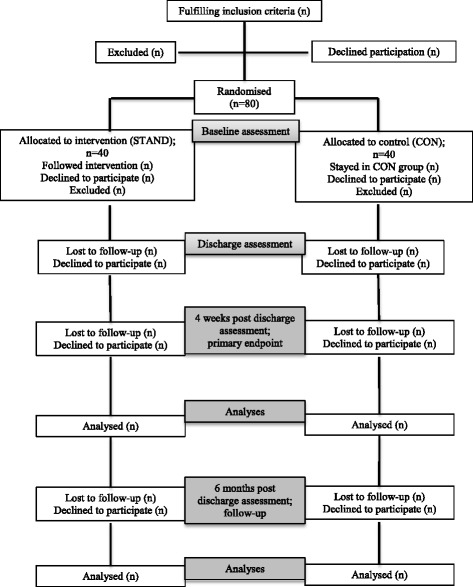
Expected flow of patients

After inclusion, baseline assessments will be performed whereafter the patients will be randomized to either: (1) intervention: progressive strength training during hospitalization and the first month after discharge (home-based), or (2) control: usual care. Shortly after discharge, four weeks (primary end point) and 6 months (follow-up) after discharge the patients will be assessed in their own homes. Figure 1 shows the study flow.

### Randomization

Patients who consent to participate will be randomly allocated to either of the two groups. Randomization will follow a computer-generated block randomization list produced by the study coordinator (JP). Randomization is stratified within the two participating municipalities. The recruitment will follow a 2:1 allocation in one of the municipalities (A) and a 1:2 allocation in the other municipality (B). This randomization procedure is followed to comply with the capacity of the municipalities (the number of physiotherapists available).

### Blinding

To ensure concealment of allocation, a study nurse will be in charge of the randomization procedure following the randomization list which will not be available to the investigators. If a patient is randomized to the intervention group, the study nurse will inform the involved physiotherapists about this allocation. Patients will be asked not to reveal to the investigators to which group they belong. The discharge test in the patient’s home will be conducted before the first post-discharge training session to avoid the investigator seeing the exercise equipment in the home. Moreover, 4-week follow-up assessments will take place between 4 and 5 weeks after discharge, and the study nurse will inform the investigators when patients are ready for the 4-week follow-up assessment (end of intervention) regardless of allocation, to avoid the investigators guessing who belongs to which group. Also, all training equipment will be removed from the patients’ homes before the 4-week assessment to ensure that the investigators do not see the equipment in those homes. Additionally, all contacts with physiotherapists in the hospital and in the municipalities regarding allocation, questions about the protocol and other practicalities will be undertaken by the study nurse to ensure blinding of the investigators. In the case of a possible adverse event and the unavailability of one of the co-authors to evaluate the severity of the event, the allocation of the patient can be revealed to the investigators in order to assure proper treatment of the patient. If the patient can no longer participate in assessments he/she will be excluded from the study. Otherwise, the patient will remain in the study and all information of all such events will be reported in the manuscript.

### Sample size

Based on data from an unpublished cohort study performed at Hvidovre Hospital, 25 consecutively included older medical patients had a mean change in the de Morton Mobility Index (DEMMI) [62] score of 1.8 and a standard deviation of 12.8 from admission to 1 month after discharge. A change of 10 points in the DEMMI score is considered a minimal clinically important difference in acute older medical patients [62]. To be able to detect a 10-point difference in the between-group change in the DEMMI score at the 4-week assessment (primary end point), we will need a sample size of 27 patients per study arm to obtain a type I error rate of 5 % and a power of 80 % for a two-sample *t* test of a normal mean difference with a two-sided significance level. We will continue to recruit patients until 54 patients have been assessed for the primary end point (4 weeks). In case of a skewed distribution of patients in the two groups (intervention and control), we will recruit until both groups contain 25 patients. We expect a maximum of 80 patients to be included in the study.

### Study principles

The protocol follows the SPIRIT 2013 (Standard Protocol Items: Recommendations for Interventional Trials) checklist [63] and the description of the intervention follows the Template for Intervention Description and Replication (TIDieR) checklist [64]. The reporting of the study once completed will follow the CONSORT (Consolidated Standards of Reporting Trials) Statement, using the extension for non-pharmacological trials [65].

### Study groups

#### Control group

Patients in the control group will receive routine care during hospitalization and after discharge. No efforts will be made to change this care during the study period. Routine care will be used as a comparator to reflect the current care for these patients.

According to the Danish Healthcare Quality Program (DDKM) [66], the functional level and nutritional status of hospitalized patients must be described within 24–48 h after admission [67] and treatment planned accordingly. No standard involves in-hospital training [67], but patients needing recovery (e.g. rehabilitation) should be identified [68], and rehabilitation (including exercise) should be planned to target the patient’s impairment and limitations. Often rehabilitation starts during hospitalization, and if it continues after discharge a rehabilitation plan must be prepared by the hospital. At Hvidovre Hospital around 5 % of older medical patients are discharged with a rehabilitation plan (personal communication with geriatric team in the ED, 30 October 2015) involving exercise therapy supervised by physiotherapists.

### Intervention group

Patients in the intervention group will receive 1:1 supervised progressive strength training daily on weekdays during hospitalization and three times per week for 4 weeks (12 training sessions, 1:1 supervised) after discharge. To account for possible cancellations, i.e. due to illness or other obstacles for training completion, distributing the 12 in-home training sessions over a maximum of 5 weeks will be allowed. The training will take place in the patient’s bedroom during hospitalization and in the patient’s own home after discharge.

#### Training intervention

All training sessions will be supervised by a skilled physiotherapist. Two physiotherapists with 3 years of experience will supervise the in-hospital training sessions and five physiotherapists with 4–15 years of experience will supervise the at-home sessions. Physiotherapists working on the medical wards of the hospital and physiotherapists involved in geriatric rehabilitation in the involved municipalities were offered participation in the study. All involved physiotherapists volunteered to participate and were granted the time needed to supervise the exercise sessions during working hours. In every training session, the patient will be asked to perform a warm-up program consisting of seated exercises for the lower extremities (hip flexions, knee extensions, heel raises, hip abductions/adductions). The patient will be asked to perform each exercise for 20 repetitions. The warm-up program has a duration of 5 min.

**Fig. 2:**
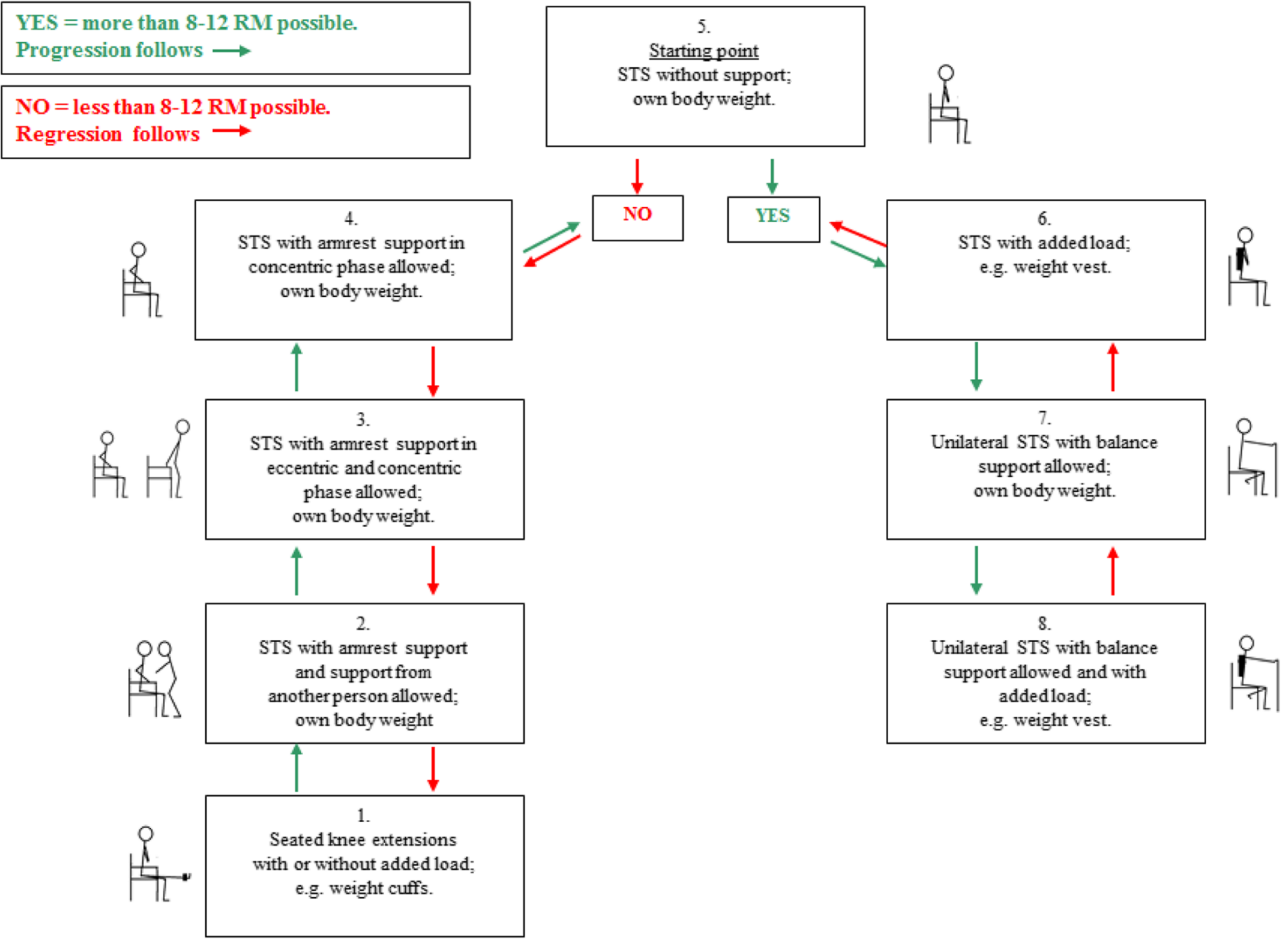
Progression model for loaded sit-to-stand exercise (STAND). *STS* sit-to-stand, *8–12 RM* 8–12 repetitions maximum (a zone in which muscular fatigue should be reached)

**Fig. 3:**
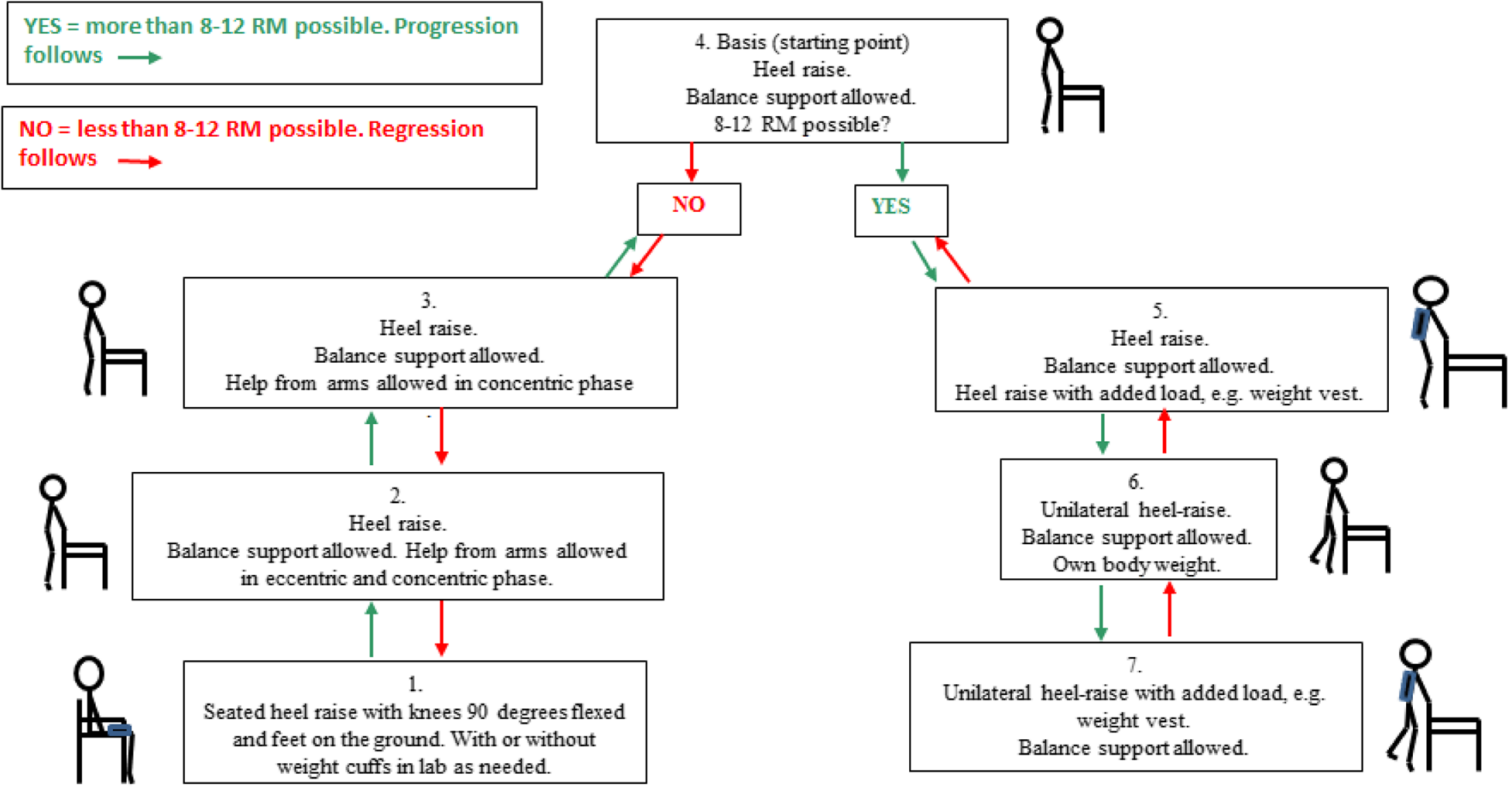
Progression model for loaded heel raise. *8–12 RM* 8–12 repetitions maximum (a zone in which muscular fatigue should be reached)

After warm-up, the patient will be asked to perform a progressive strength training program for the lower extremities, based on a minimum treatment approach, consisting of a sit-to-stand exercise (Fig. 2) and a heel raise exercise (Fig. 3) as outlined in detail below. For both exercises the progression will follow pre-defined models based on the STAND model (Fig. 2), which we have tested and found feasible in older medical patients [61]. In each exercise, the progression model allows for performing the exercise from a seated position (level 1) to performing the exercise unilaterally with extra load added (level 7/level 8). The patient will be asked to perform three sets of 12 repetitions maximum (RM) of each exercise. This will correspond to 60–70 % of 1 RM [30, 69, 70]. The aim will be to reach contraction failure (muscular fatigue) at a relative load zone of 8–12 RM in each set [30]. A 2-min pause will be allowed between sets [30]. The correct level of each exercise will be chosen according to the progression models by the supervising physiotherapist. The patient will be asked to work at moderate velocity taking 2 s for the concentric (raising) phase and 2 s for the eccentric (lowering) phase of the exercise. An isometric pause of 1 s will be allowed after both the concentric and the eccentric phase [30]. If a patient can perform six non-compensatory repetitions and needs a little support performing the last repetitions (e.g. minimal use of armrests/minimal balance support), and if a proper technique is maintained, training at the given level will be accepted to enable the patients to reach fatigue in every set. Moreover, increased speed will be allowed in the last two repetitions of each set to optimize leg power, which has been shown to be associated with physical performance in mobility-limited older adults [54, 71]. Each set of each exercise is considered unique and determines whether the patient will stay on the same level or either progress or regress. The total duration of each exercise session will be approximately 10–15 min.

#### STAND

Each training session will begin with the sit-to-stand exercise. The patient will be asked to sit in a standard chair with armrests with a seat height of approximately 45 cm. The chair is placed so that it cannot slide during the exercise. The patient is to keep the feet on the floor at shoulder width and to cross the arms at the wrists with the hands placed on the opposite shoulder. The starting point in STAND is level 5 (Fig. 2). The patient will be asked to rise to a fully extended position and to sit down at a constant pace and will be verbally encouraged by the supervising physiotherapist to perform as many repetitions as possible, maintaining the same pace to ensure training to contraction failure [69]. If the patient is able to perform more than 12 repetitions he/she will progress to the next level (level 6), performing the exercise wearing a weight vest (Titan Box, 1–30 kg) containing the number of kg required to reach 8–12 RM, and so forth. If the patient is not able to perform eight repetitions at level 5, regression is permitted (to level 4) allowing the patient to use the armrests in the concentric phase, and so on.

#### Heel raise

The progression of the heel raise exercise will follow the progression model for heel raise (Fig. 3). The patient will be asked to stand behind a standard chair keeping the hands lightly on the back of the chair for balance support. The patient is asked to keep the feet on the floor at shoulder width. The starting point in the progression model is level 4 (Fig. 3). The patient will be asked to lift both heels to stand on the forefeet and to lower their heels to a standing position at a constant pace. The patient will be verbally encouraged by the physiotherapist to perform as many repetitions as possible, maintaining the same pace to ensure training to contraction failure [69]. If the patient is able to perform more than 12 repetitions he/she will progress to the next level (level 5), performing the exercise wearing a weight vest (Titan Box, 1–30 kg) containing the number of kg required to reach 8–12 RM, and so forth. If the patient is not able to perform eight repetitions at level 4, regression is permitted (to level 3) allowing the patient to use the back of the chair as support in the concentric phase, and so forth.

#### Protein supplement

In this study, protein is considered as an integral part of strength training to optimize the anabolic response after training. Therefore, immediately after each training session the patient will be asked to consume an oral protein supplement (Nutridrink Compact Protein from Nutricia A/S) containing 18 g milk-based protein and 300 kcal.

### Standardization of intervention

The primary investigator will perform pre-intervention meetings with all involved physiotherapists to ensure standardization of the intervention. At the meetings, the physiotherapists will be introduced to the warm-up program and the strength training protocol. At the meeting, the strength training exercises will be performed by all involved physiotherapists to ensure common knowledge about the requirements at each level of the program. A laminated version of the warm-up program as well as the progression models for both strength training exercises will be provided to all involved physiotherapists. During the study period, the physiotherapists will be able to contact the primary investigator or a study nurse at all times should any questions arise. If a physiotherapist leaves the project, e.g. in the case of leave of absence or ending employment, the primary investigator will ensure that the physiotherapist taking over will be introduced to the protocol in the same manner as the physiotherapists who are already involved.

### Outcome measures

Outcomes measures will be assessed on admission (baseline), shortly after discharge, approximately 4 weeks after discharge (primary end point) and 6 months after discharge. All outcomes to be assessed are presented in Table 2.

**Table 2:** Variables to be assessed

Variable	Baseline	Discharge	4 weeks	6 months
Primary outcome				
de Morton Mobility Index (DEMMI)	+	+	+	+
Secondary outcomes				
24-h mobility (*activ*Pal3™ monitors; 1-week assessments)	+	+	+	+
Isometric knee extension strength	+	+	+	+
30-sec sit-to-stand test	+	+	+	+
Habitual gait speed (HGS)	+	+	+	+
Hand-grip strength (HG)	+	+	+	+
Activities of Daily Living (Barthel Index 20)	+	+	+	+
Descriptive variables				
Age	+			
Gender	+			
Weight	+	+	+	+
Educational level	+			
Living status	+	+	+	+
History of smoking	+			
Use of ambulatory devices	+	+	+	+
Use of municipal help	+	+	+	+
History of falls during the last year	+	+	+	+
Falls Efficacy Scale	+	+	+	+
Nutritional Risk Screening (NRS)	+	+	+	+
New Mobility Score (NMS)	+	+	+	+
Cumulated Ambulation Score (CAS)	+	+	+	+
Days per week spent outdoors	+	+	+	+
Hospitalization within last 4 weeks			+	
Hospitalization within last 6 months	+			+
Possible confounders and modifiers				
Age	+			
Gender	+			
Cognition				
Short Orientation-Memory-Concentration test (OMC)	+			
Mini Mental State Examination (MMSE)		+	+	+
Trail Making Test (Trails)		+	+	+
Digit Symbol Substitution Test (DSST)		+	+	+
Hopkins Verbal Learning Test (HVLT)		+	+	+
Geriatric Depression Scale (GDS)		+	+	+
Health status (EQ-5D)	+	+	+	+
Self-rated health (EQ-5D)	+	+	+	+
Mini Nutritional Assessment (MNA)	+	+	+	+
Self-reported physical activity	+	+	+	+
Verbal Ranking Scale (VRS)	+	+	+	+
Medications	+	+	+	+
History of training before hospitalization	+			
History of training in the municipality after discharge		+	+	+

### Primary outcome measure

The primary outcome will be change in the DEMMI score from baseline to 4 weeks after discharge (end of intervention, primary end point). The DEMMI is a valid and reliable measure of mobility in both acute and subacute older medical patients and in community-dwelling older adults [62, 72–74], and can be used to accurately monitor mobility in older adults [72]. It includes observations ranging from mobility to dynamic balance and is scored on a scale from 0 to 100 with 100 representing the highest level of mobility [72, 73], and with a minimal clinically important difference of 10 points for acute older medical patients [72].

### Secondary outcome measures

Secondary outcomes will be the following six: (1) 24-h mobility measured by an *activ*PAL3™ activity monitor (PAL Technologies Ltd., Glasgow, UK). The patient will be asked to wear an *activ*PAL3™ on the thigh during hospitalization, the first week after discharge, the first week after the 4-week assessment and the first week after the 6-month assessment. The patient will wear the *activ*PAL3™ halfway between the spina iliaca anterior superior and the patella on the front side of the right thigh. The monitor will be covered in Tegaderm™ transparent waterproof film (3 M, Maplewood, MN, USA), attached to the patient by a PAL*stickie*™ (dual-layer hydrogel adhesive pad) and covered by Leukomed® T transparent film (BNS medical, Hamburg, Germany) to enable the patient to wear the *activ*PAL3™ while showering. The patient will be asked to wear the monitor for 24 h per day. The *activ*PAL3™ can record continuously for 7 days, whereafter the monitor will be replaced should the hospitalization be of a longer duration. The *activ*Pal3™ accelerometer measures time spent sitting/lying, standing and walking, the number of steps taken, cadence and the number of sit-to-stand and stand-to-sit transitions. The *activ*Pal3™ is a valid and reliable measure of posture and transitions in healthy young and mobility limited older adults [75–77] and of walking at speeds between 0.67 m/s and 1.56 m/s in young and older adults [78–80]. Unpublished data from Hvidovre Hospital regarding 317 older medical patients has shown that 46 % of them walked at speeds below 0.67 m/s, why time spent walking could potentially be categorized as standing for 46 % of older medical patients. For this reason, if 15 % of the total sample walk at speeds below 0.67 m/s the *activ*Pal3™ data will be dichotomized into sedentary (sitting/lying) and upright time (walking/standing); (2) isometric knee extension strength (IKE) in the dominant leg using a handheld dynamometer (Power Track II Commander; JTech Medical, Midvale, UT, USA). The patient will be seated in a standard chair with a seat height of approximately 45 cm, with the arms crossed over their chest and 90° knee flexion [81, 82]. A strap will be attached to the chair and the patient’s ankle, just proximal to the malleoli. A transducer will be placed under the strap and a thin foam pad will be placed between the transducer and the leg. The distance between the lateral femoral epicondyle and the center of the transducer will be measured (the moment arm). The patient will be asked to extend the leg as forcefully as possible for 5 s three times with a 1-min pause in between. Up to two additional contractions will be performed if the last contraction elicits the highest value to ensure that maximal force is measured. Isometric knee extension strength will be expressed as maximal force (Nm) per kg body weight (kg); (3) the 30-sec sit-to-stand test (STS) using a standard armchair with a seat height of 45 cm [83]. The patient will be asked to sit with the arms crossed over the chest and to stand up once without using the arms. If this is performed safely, the patient will be asked to stand up fully and sit down as many times as possible in 30 s with the arms across the chest. The number of full stands will be counted. If the patient is not able to rise once from the chair without using the arms, a modified STS will be used, allowing the patient to use the armrests for support; (4) habitual gait speed (HG) on a 4-m course [84, 85]. The patient will be asked to walk 4 m at usual pace starting from a standing position. A walking aid will be allowed if needed. The faster of two walks will be used as the outcome; (5) hand-grip strength (HGS) in the dominant hand using a handheld dynamometer (Digi-II; Saehan). The patient will be placed in a sitting position in an armchair, with the lower arm placed on the armrest, an elbow flexion of 90° and the wrist in a neutral position. The patient will be asked to place the contralateral hand on the leg with the palm facing upwards. The dynamometer handle will be set at position 2 [86] and the investigator will reset the dynamometer before handing it to the patient and will ask the patient to squeeze the handle as forcefully as possible for 5 s. The patient will be asked to perform the test three times with a 1-min pause in between. If the third test shows the highest value additional tests will be performed until performance of a lower value to ensure that the highest value possible is obtained. Hand-grip strength will be expressed in kg; (6) the Barthel Index 20 (BI) is used as a measure of Activities of Daily Living (ADL) [87]. The BI assesses the help needed in regard to grooming, toilet use, feeding, transfer, mobility, dressing, stair climbing and bathing, and in addition the presence or absence of urinary and fecal incontinence. A score of between 0 and 20 can be obtained with higher scores indicating less disability.

### Additional variables

Descriptive variables and possible confounders and modifiers for exploratory analyses will be collected. Descriptive variables will include: education, living status, history of smoking, use of ambulatory devices, use of municipal help, history of falls during the last year, Nutritional Risk Screening (NRS) [88–90], the New Mobility Score (NMS) (recall of mobility 2 weeks before admission and on the day of admission) [91, 92] and the Cumulated Ambulation Score (CAS) [93]. Possible confounders and modifiers will be assessed: gender; age; cognition by the Short Orientation-Memory-Concentration test (OMC) [94], the Mini Mental State Examination (MMSE) [95], the Trail Making Test (Trails) [96, 97], the Digit Symbol Substitution Test (DSST) [98], and the Hopkins Verbal Learning Test – Revised (HVL-R) [99, 100]; depression by the Geriatric Depression Scale (GDS) [101]; health status by the EuroQol instrument (EQ-5D) [102]; nutritional state by the Mini Nutritional Assessment (MNA) [103]; self-reported physical activity by a four-level questionnaire [104, 105]; pain before and after training by the Verbal Ranking Scale (VRS) [106, 107]; medications, history of training before admission, and history of training in the municipality after discharge. Moreover, baseline level of DEMMI and 24-h mobility using assessments from the first week after discharge will be treated as possible confounders and modifiers. Based on the cognitive assessments, patients will be categorized as having mild cognitive impairment (MCI) or not, and those with MCI will be further sub-categorized as amnestic-MCI, non-amnestic MCI or multiple-MCI [108]. These categories will be used in the analyses.

### Data collection

The primary investigator and a team of three assistant investigators will perform all baseline and follow-up assessments. All four investigators are trained physiotherapists with 1 to 15 years of experience.

The admission assessments will be performed on the acute medical admissions ward or on an internal medicine ward at Copenhagen University Hospital, Hvidovre, Denmark, within the first 48 h after admission. All follow-up assessments will be performed in the patient’s own home, and the same investigator will assess the same patient at all assessments whenever logistically possible, to promote patient retention.

During each training session the supervising physiotherapist will complete an exercise diary consisting of information about the level of exercise attained according to the progression models, the extra load added (kg), and the number of sets and repetitions performed at each level. Self-reported pain will be registered immediately before and after each training session by the use of the VRS. Moreover, the physiotherapist will register reasons for non-participation as well as the amount of protein consumed after each training session. The patient time line including data collection is presented in Table 3.

**Table 3:** Patient timeline

	Study period						
Time point	Admission	Baseline	Hospital intervention	Discharge assessment	Home intervention	4-week assessment	6-month assessment
		≤48 h after admission	Daily during hospitalization	In patient’s home	3 times per week for 4 weeks in patient’s home	In patient’s home	In patient’s home
Enrollment							
Eligibility screen	X						
Informed consent	X						
Study groups							
Strength training		X	X	X	X	X	X
Control		X		X		X	X
Assessments							
Baseline assessment^a^		X					
Primary outcomes		X		X		X	X
Secondary outcomes		X		X		X	X
Descriptive variables and possible confounders and modifiers		X		X		X	X

^a^See Table 2 for a detailed description of assessed variables

### Compliance

High compliance with the intervention is defined as completion of 80 % of all training sessions with a minimum of two sets performed per session.

### Data management

All case report forms will be checked for errors and missing data before being archived in a study database and all paper-based versions will be locked in a filing cabinet to ensure confidentiality. The primary investigator will have access to the full dataset, in which no information about allocation is visible, and co-investigators will have access as needed. Data management will comply with the rules of the Danish Data Protection Agency. The full protocol will be published, and public access to de-identified patient-level data will be provided once the data have been analyzed. All data will be double entered in Epidata Entry 3.1 (Epidata Associations, Odense, Denmark), range checked for data values, checked against the paper-based assessments and exported to SAS Enterprise Guide 6.4 (SAS Institute Inc., Cary, NC, USA). Data from the *activ*PAL3™ will be downloaded to a computer using *activ*PAL™ Professional Software, version 7.2.32. For each *activ*PAL3™ monitoring the investigators will note the time and date that the monitor is attached to the patient, the time and date that monitoring is started, when the monitor is removed from the patient, and reasons for not wearing the monitor if it is removed prematurely.

### Statistical analyses

#### Descriptive data

Descriptive data for the intervention and control groups will be compared using the chi-square test for categorical variables, the Student’s *t* test for normally distributed continuous variables, and the Mann-Whitney *U* test for non-parametric variables. Descriptive data will be presented as means with standard deviations, medians with inter-quartile ranges or frequencies with percentages depending on the distribution of the variable.

### Primary analysis for the primary outcome

A mixed-model analysis (dif (discharge-baseline), dif (4 weeks-baseline), dif (6 months-baseline)) will be performed using the SAS procedure PROC MIXED. The patient identification number and municipalities will be modeled as random variables, and both group and time will be modeled as fixed factors. The between-group difference in change in DEMMI will be estimated from the interaction between the time and group variable. The primary outcome will be the between-group difference in change in the DEMMI score from baseline to 4 weeks after discharge (end of intervention). The primary analysis will follow the intention-to-treat principle using multiple imputation in case of missing outcome measures and will be unadjusted.

### Secondary and supplementary analyses

From the primary analysis model, the effect during hospitalization and the post-intervention effect (change from 4 weeks to 6 months post discharge) will be estimated. For the secondary outcomes, similar analyses will be performed. Moreover, all analyses will be using adjustments for baseline DEMMI. To account for imbalances in in-hospital time, a sub-analysis will be performed for the effect during hospitalization and the effect from baseline to end of intervention adjusted for length of stay. Additionally, the unadjusted repeated model will be carried out following the per protocol principle, comparing patients who have fulfilled the compliance criteria with the control group. All between-group differences will be expressed as the average difference in change from baseline. The analyses outlined above will all be reported in the main trial manuscript regarding effect of the strength training program.

If additional funding is obtained, more patients will be included to increase the sample size to obtain sufficient power to model statistical interactions and perform secondary analyses (described below), which will be published subsequently. To investigate the possible influence of confounders and modifiers on the effect of the intervention on DEMMI, an unadjusted analysis of variance of the between-group change from baseline to 4 weeks post discharge in the DEMMI score will be performed. In addition, this model will be extended by adjusting for all and each of the potential confounders and modifiers one by one. Confounding effects will be evaluated by comparing the unadjusted effect of group with the adjusted effects. Moreover, to investigate whether or not the effect of the intervention is modified by the potential confounders and modifiers the adjusted models will be extended with an interaction term between group and the potential confounders and modifiers. Similar analysis will be performed with 24-h mobility (average time spent standing or walking per 24 h) and with those of the secondary outcomes that showed a significant between-group difference in the primary analysis. Also, a logistic regression with compliance as the outcome and each of the potential confounders and modifiers as covariates will be performed. All analyses will follow both the intention-to-treat and the per protocol principle.

To investigate the effect of the intervention on cognition (MCI status, MMSE, OMC, HVLT, DSST, Trails A and B) at 4 weeks, the following analyses will be performed. A generalized logistic regression for MCI status and an analysis of variance for MMSE, OMC, HVLT, DSST, and Trails A and B will be used with group as the independent variable. The analyses will follow the intention-to-treat principle with multiple imputation for missing values. Moreover, these analyses will also be performed adjusting for baseline OMC, baseline DEMMI, gender and age, depression, health status, nutritional state, self-reported physical activity, pain, medications, length of stay and 24-h mobility using assessments from the first week after discharge, and possible interactions with group will be analyzed. Additionally, the models will be repeated following the per protocol principle comparing patients. All results will be expressed as estimated means’ differences between the intervention and control group with the corresponding 95 % confidence intervals.

All models will be investigated for goodness-of-fit (linearity, variance homogeneity and normal distribution of residuals) by visual inspection of plots and remodeling will be performed accordingly. All statistical tests will be performed using SAS (SAS Institute Inc., Cary, NC, USA) and *p* values ≤0.05 will be considered statistically significant. However, for all analyses evaluating potential modifiers and confounders of the intervention *p* values ≤0.01 will be used to account for multiple testing. No interim analysis will be made.

### Publication process

MMP will ensure that the results of the study are published in due time after study termination. The reporting of study will follow the CONSORT extension for randomized trials of non-pharmacological trials [65].

### Changes to initial plan

In the statistical analysis plan, imputation for missing data was changed from “last observation carried forward” to “multiple imputation.” This protocol change was made before inclusion was completed, and while the study was still blinded. The BI was added as a secondary outcome before inclusion of the first patient to enable comparison with previous studies evaluating ADL during and after hospitalization [9, 10, 17, 109].

From February 2014, patients have been included from an additional municipality, the municipality of Hvidovre, due to the possibility of providing in-home training for these patients as well. Randomization in this municipality follows a 2:2 allocation. From September 2014, patients assigned to physical rehabilitation in the community have no longer been excluded from the study, as rehabilitation in the community is rarely commenced until 4 weeks after discharge and thus after the study’s primary end point. From January 2015, a physician has performed the initial screening of all eligible patients and informed the patients about the study before referring them to the primary investigator for informed consent and baseline assessments to enhance enrollment.

### Ethics

The patients will be informed that participation is voluntary and that they can withdraw at any time without losing their right to treatment. The study is approved by the Ethics Committee of the Capital Region of Denmark (H-2-2012-115) and by the Danish Data Protection Agency (2007-58-0015) and is registered at ClinicalTrials.gov (NCT01964482). The Ethics Committee will be informed about important protocol modifications for approval.

## Discussion

There is limited data on the effect of strength training initiated during hospitalization and continued after discharge in older medical patients, and details about the optimal nature and dose of exercise are needed [29, 50, 51]. Higher intensities seem superior to lower intensities in older adults [52, 110, 111], and supervision is essential for compliance [49]. This study provides a detailed description of a simple, supervised cross-continuum strength training program, based on a minimal time-consuming treatment approach. All physiotherapists involved are thoroughly instructed in the intervention to try to obtain standardization and avoid a cluster effect. The treatment approach was chosen to investigate whether as few as two well-performed strength training exercises per session, combined with protein supplementation, during hospitalization and 4 weeks after discharge, can improve mobility in older medical patients. This approach was chosen to facilitate implementation in a busy clinical care setting, given a positive trial outcome.

### Data monitoring

No data committee will be established as the intervention is considered to be low-risk. All investigators and physiotherapists will be asked to report adverse events to MMP, and the study will be stopped if the adverse event is considered to be caused by training or testing. A “hotline” to an ED geriatrician has been established should an adverse event occur or should the investigators need advice regarding a patient. The authors will meet frequently during the study to discuss trial conduct.

### Roles and responsibilities

The study has been designed at Optimed, Clinical Research Centre, Copenhagen University Hospital, Hvidovre, Denmark. The trial is overseen by the group of authors.

### Study status

Recruitment of patients is ongoing at the time of submission of this protocol. Recruitment began in October 2013 and is expected to end in March 2016.

## Abbreviations

ADL: Activities of Daily Living
BI: Barthel Index 20
CAS: Cumulated Ambulation Score
CONSORT: Consolidated Standards of Reporting Trials
COPD: chronic obstructive pulmonary disease
DDKM: Danish Healthcare Quality Program
DEMMI: de Morton Mobility Index
DSST: Digit Symbol Substitution Test
ED: Emergency Department
GDS: Geriatric Depression Scale
HG: habitual gait speed
HGS: hand-grip strength
HVLT-R: Hopkins Verbal Learning Test – Revised
IKE: isometric knee extension strength
MCI: mild cognitive impairment
MMSE: Mini Mental State Examination
MNA: Mini Nutritional Assessment
NMS: New Mobility Score
NRS: Nutritional Risk Screening
OMC: Short Orientation-Memory-Concentration test
RM: repetitions maximum
SPIRIT: Standard Protocol Items: Recommendations for Interventional Trials
STS: 30-sec sit-to-stand test
TIDieR: Template for Intervention Description and Replication
Trails: Trail Making Test
VRS: Verbal Ranking Scale
